# Supplementary material for: Cell-type-resolved genetic regulatory variation shapes inflammatory bowel disease risk
Source: medRxiv. 2025 Jun 24:2025.06.24.25330216. Preprint. [Version 1] doi: 10.1101/2025.06.24.25330216 (PMC12262763; doi:10.1101/2025.06.24.25330216)
Supplement: 1 [file NIHPP2025.06.24.25330216V1-supplement-1.pdf]

medRxiv preprint doi: https://doi.org/10.1101/2025.06.24.25330216; this version posted June 25, 2025. The copyright holder for this preprint (which was not certified by peer review) is the author/funder, who has granted medRxiv a license to display the preprint in perpetuity.
It is made available under a CC-BY 4.0 International license .

1 Supplementary figures

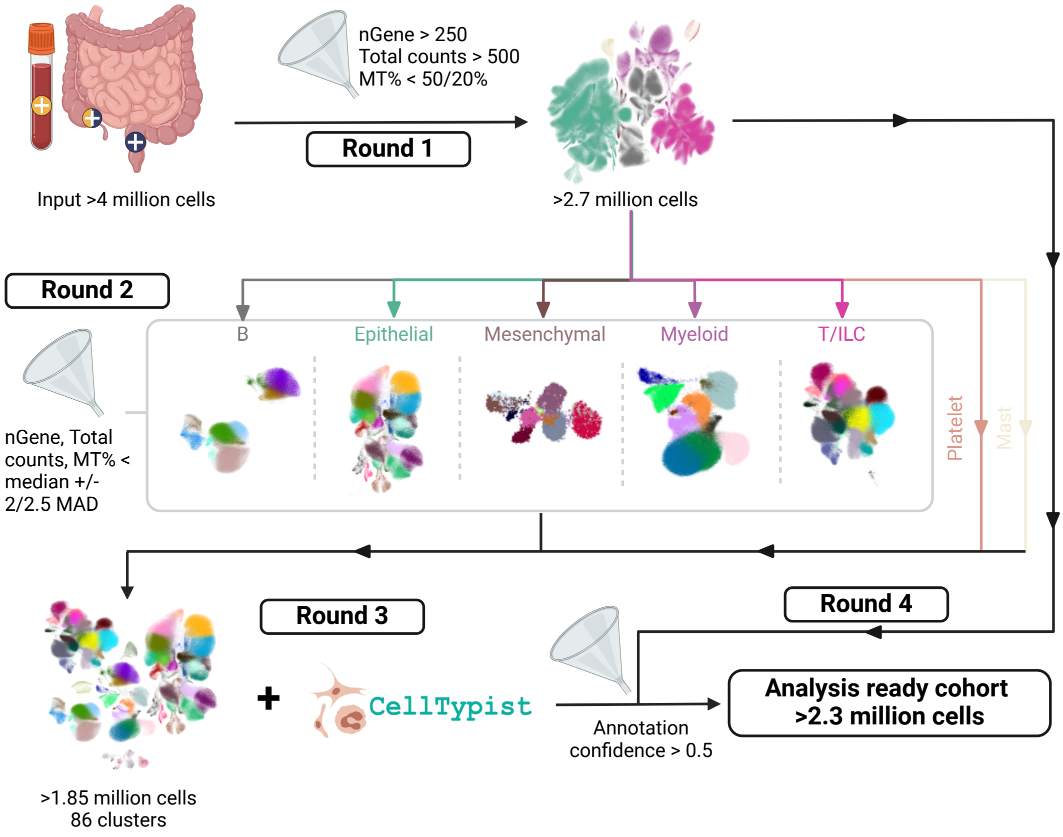

Supplementary Figure 1: **Overview of quality control, integration and clustering approach for scRNAseq data.** Analysis was performed across 4 rounds, a detailed summary of which is available in Methods. Cells were first filtered by basic quality control, integrated and clustered at a coarse resolution to identify broad lineages (see Supplementary Figure S12). These were then divided by lineage, and all except Platelet and Mast cells were subject to a second round of quality control using relative thresholds, before re-integration and clustering across a range of resolutions. The maximum resolution at which transcriptionally distinct populations of each lineage could be reproducibly obtained was then detected. Lineages were then recombined in a final round of integration, producing the total 86 cell-types. See Supplementary Figure S14 for outline of genes used to annotate individual cell-type. This was then used to derive a CellTypist model, which was used to annotate cells that passed quality control from the first round, producing the analysis-ready set. nGene = The number of genes expressed per cell, Total counts = total number of counts per cell, MT% = the percentage of counts mapping to genes in the mitochondrial genome, MAD = the number of median absolute deviations a cell's quality control parameter is from the median for that given parameter.

medRxiv preprint doi: https://doi.org/10.1101/2025.06.24.25330216; this version posted June 25, 2025. The copyright holder for this preprint (which was not certified by peer review) is the author/funder, who has granted medRxiv a license to display the preprint in perpetuity.
It is made available under a CC-BY 4.0 International license .

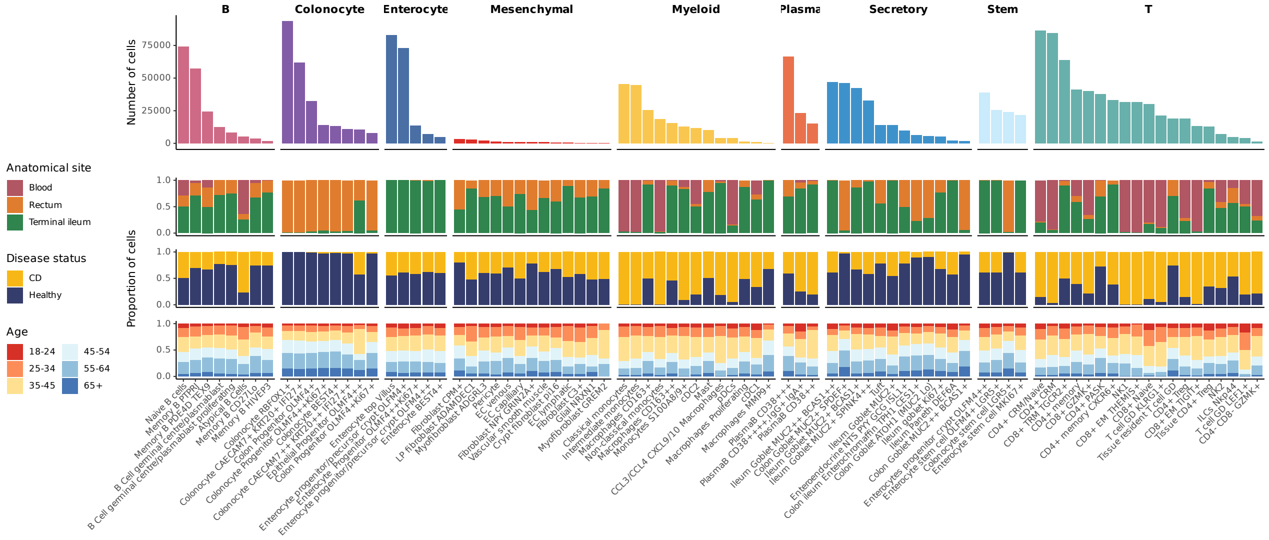

Supplementary Figure 2: **Cell-type level metadata**. Total number (top) and contribution of different sites, disease status, and ages of sample to each cell-type in the 1,837,436 cells in the atlasing cohort.

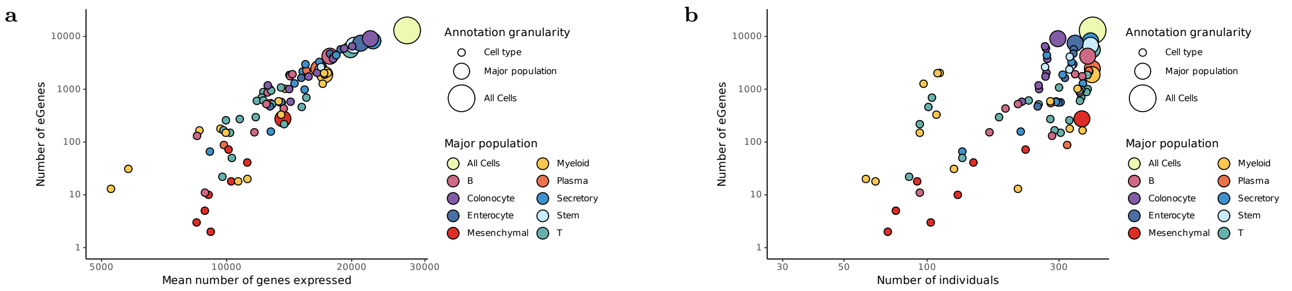

Supplementary Figure 3: **Factors influencing eGene detection.** Association between between the number of eGenes detected in the cross-site analysis and a) the mean number of genes per pseudobulked sample or b) the number of individuals.

medRxiv preprint doi: https://doi.org/10.1101/2025.06.24.25330216; this version posted June 25, 2025. The copyright holder for this preprint (which was not certified by peer review) is the author/funder, who has granted medRxiv a license to display the preprint in perpetuity.
It is made available under a CC-BY 4.0 International license .

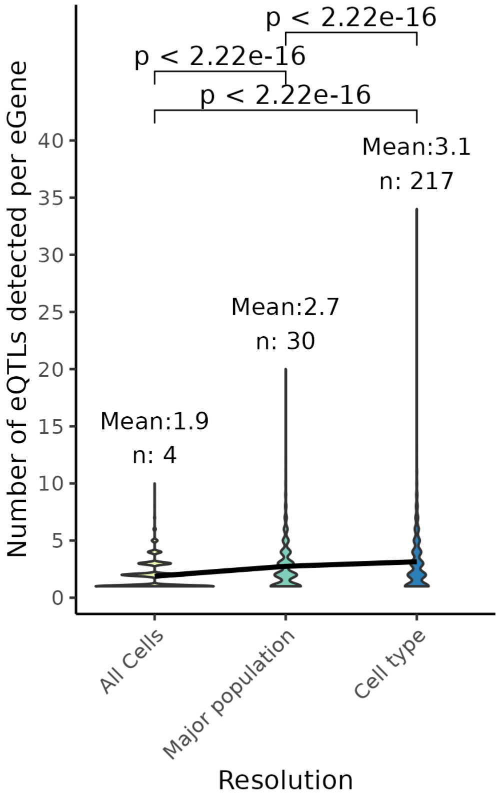

Supplementary Figure 4: **Number of eQTLs detected at each resolution.** Comparison of the number of eQTLs (detectermined by linkage disequilibrium clumping, see Methods) that were found for each eGene at each resolution. Black line connects the means of each resolution, 'n=' indicates the total number of annotations per resolution, p-values calculated by wicoxon test.

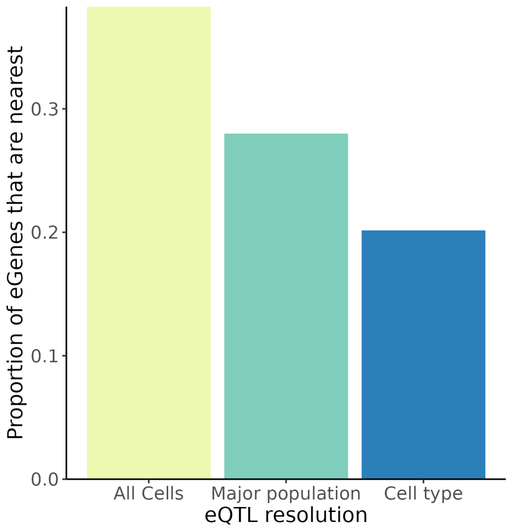

Supplementary Figure 5: **eQTLs detected at high resolutions are less frequently associated with the nearest gene.** Proportion of eQTLs detected at each resolution for which the nearest gene is the eGene.

medRxiv preprint doi: https://doi.org/10.1101/2025.06.24.25330216; this version posted June 25, 2025. The copyright holder for this preprint (which was not certified by peer review) is the author/funder, who has granted medRxiv a license to display the preprint in perpetuity.
It is made available under a CC-BY 4.0 International license .

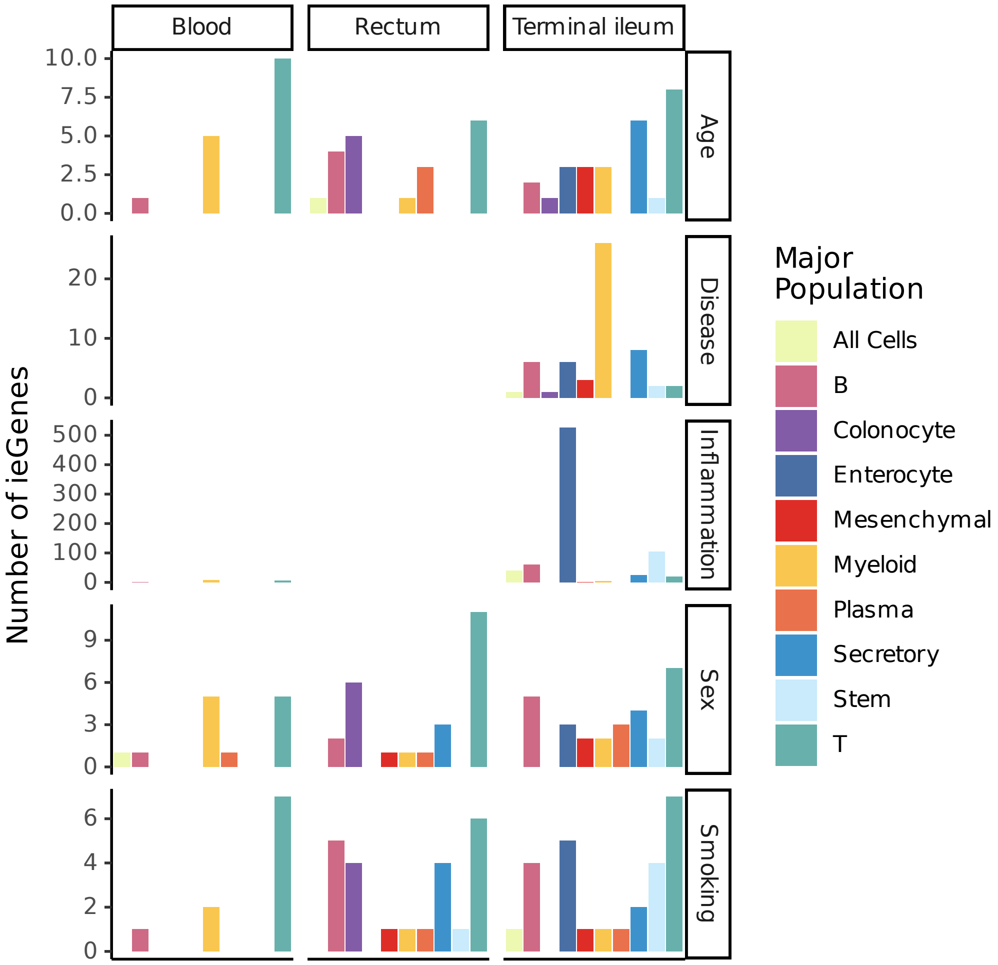

Supplementary Figure 6: **Number of ieGenes detected for each variable.** Absolute number of eGenes detected by interaction (ieGenes) with sample level phenotypes, grouped by the interaction variable and the major population in which ieGenes were detected.

medRxiv preprint doi: https://doi.org/10.1101/2025.06.24.25330216; this version posted June 25, 2025. The copyright holder for this preprint
(which was not certified by peer review) is the author/funder, who has granted medRxiv a license to display the preprint in perpetuity.
It is made available under a CC-BY 4.0 International license .

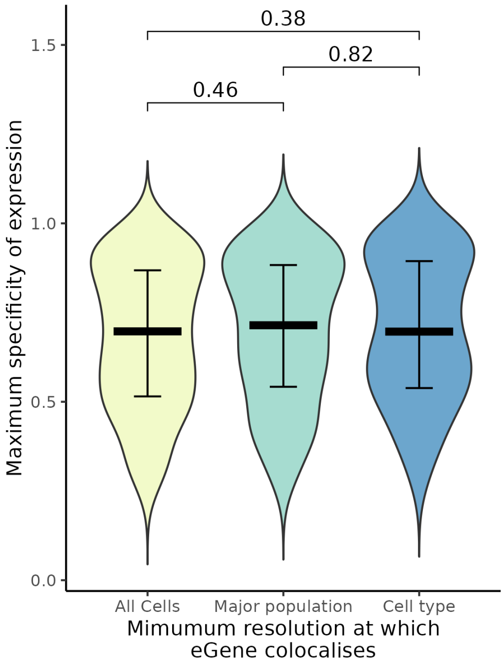

Supplementary Figure 7: **Disease effector genes identified at high resolutions are not more specifically expressed than those at lower resolutions.** The maximum value of the per-cell-type expression specificity) of disease effector genes, as identified by CELLEX (Timshel *et al.,* 2022), grouped by the minimum resolution at which the colocalisation was detected.

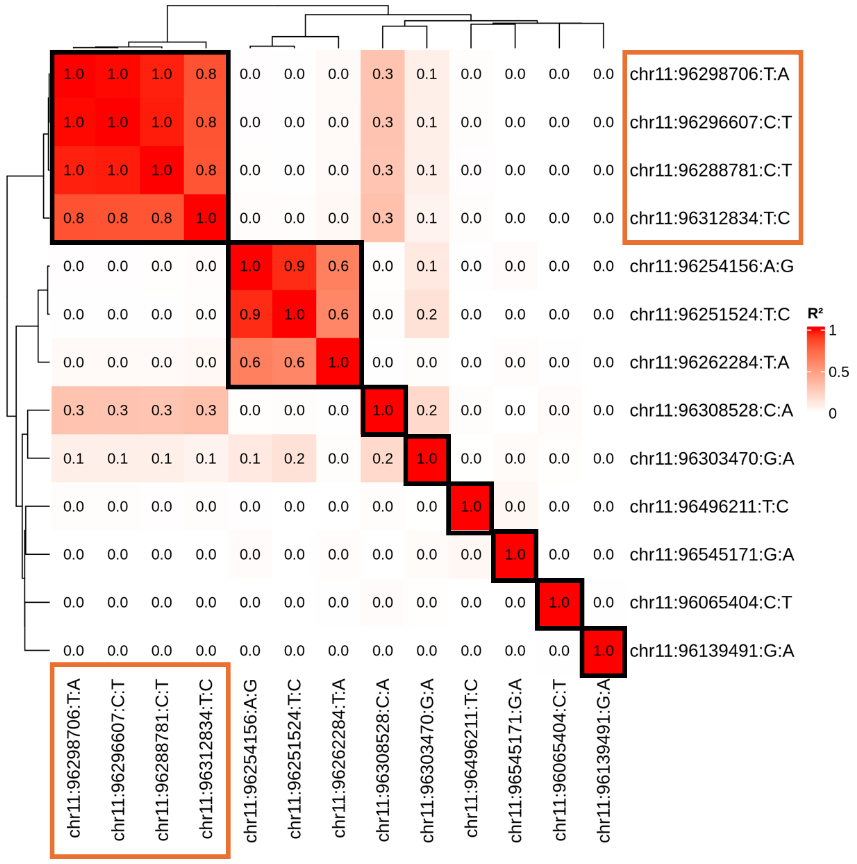

Supplementary Figure 8: **Independence of eQTLs for *MAML2* dysregulation.** Heatmap depicting the pairwise linkage disequilibrium (r^2) between lead variants from each condition with a significant eQTL. Clumped effects are indicated by black boxes around pairise tests. Lead variants that colocalise with IBD are outlined by an orange box.

medRxiv preprint doi: https://doi.org/10.1101/2025.06.24.25330216; this version posted June 25, 2025. The copyright holder for this preprint (which was not certified by peer review) is the author/funder, who has granted medRxiv a license to display the preprint in perpetuity.
It is made available under a CC-BY 4.0 International license .

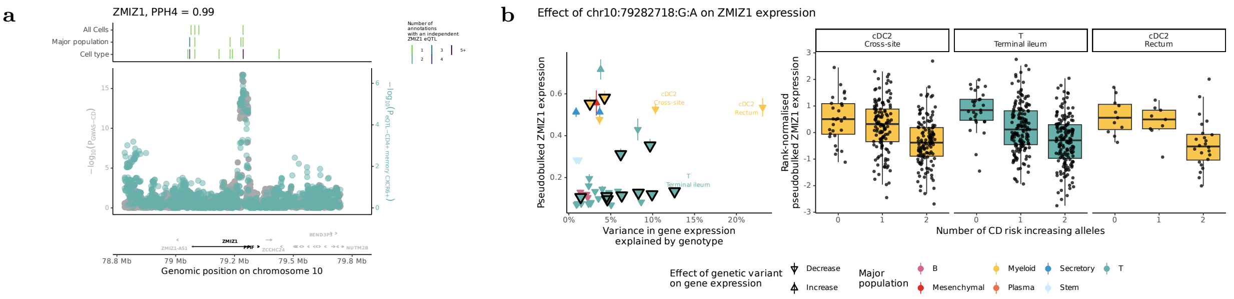

Supplementary Figure 9: **Identification and contextualisation of NOTCH signalling colocalisation target *ZMIZ1*** (a) Regional association pattern between genetic dysregulation of *ZMIZ1* and CD susceptibility. (b) Comparison of the variance of disease effector gene expression explained by the colocalising variant across conditions where a nominal ($p < 0.05$) eQTL effect was detected (left), and boxplots showing effect in the three annotations with the most variance explained (right).

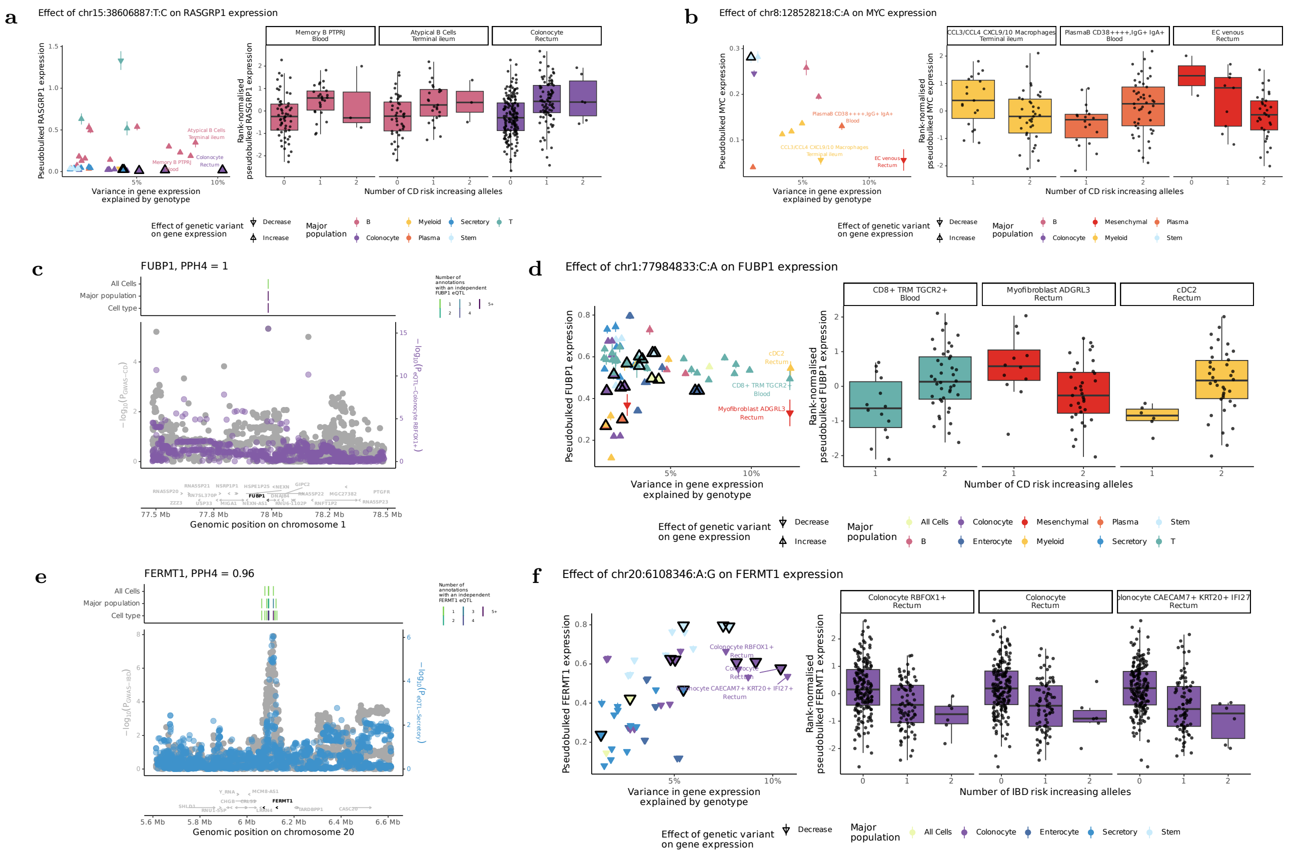

Supplementary Figure 10: **IBD colocalisations may impact epithelial barrier function and Wnt signalling**. (a/b/d/f) Comparison of the variance of disease effector gene expression explained by the colocalising variant across conditions where a nominal ($p < 0.05$) eQTL effect was detected for each gene (left), and boxplots showing effect in the three annotations with the most variance explained (right). (c/e) Regional association pattern between genetic dysregulation of *FUBP1* and CD susceptibility or *FERMT1* and IBD susceptibility, respectively.

medRxiv preprint doi: https://doi.org/10.1101/2025.06.24.25330216; this version posted June 25, 2025. The copyright holder for this preprint (which was not certified by peer review) is the author/funder, who has granted medRxiv a license to display the preprint in perpetuity.
It is made available under a CC-BY 4.0 International license .

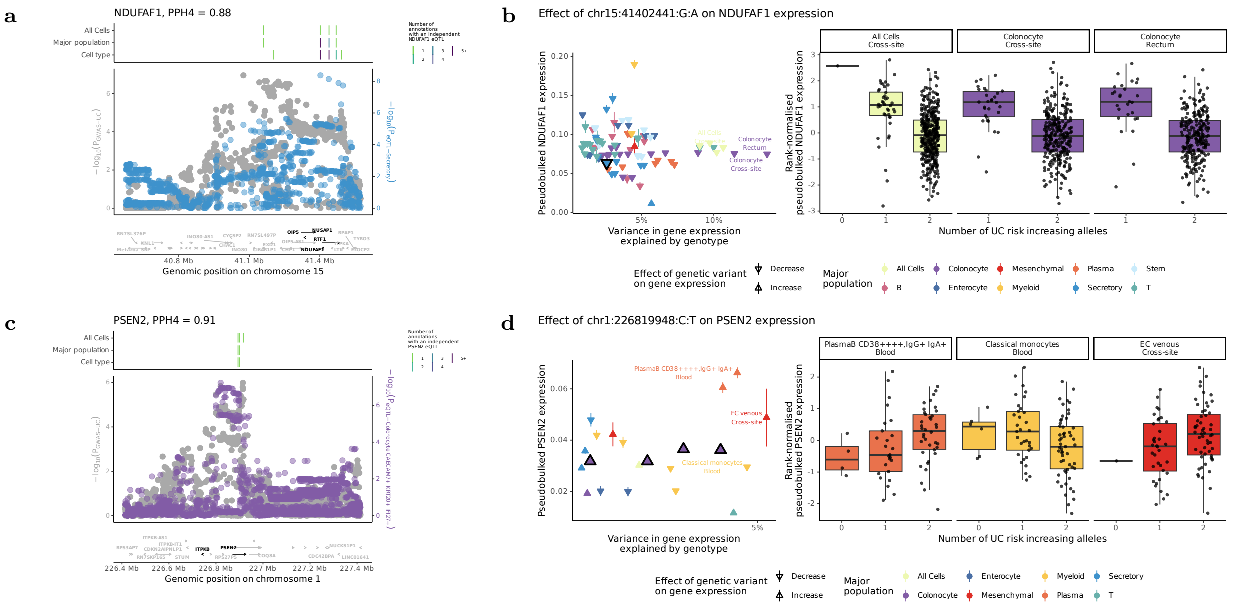

Supplementary Figure 11: **Colocalisation of UC with dysregulation of existing therapeutic targets *NDUFAF1* and *PSEN2*.** Regional association pattern between genetic dysregulation of *NDUFAF1* (a) and *PSEN2* (c) and IBD types. Comparison of the variance of disease effector gene expression explained by the colocalising variant across conditions where a nominal ($p < 0.05$) eQTL effect was detected for each gene (b/d, left), and boxplots showing effect in the three annotations with the most variance explained (right).

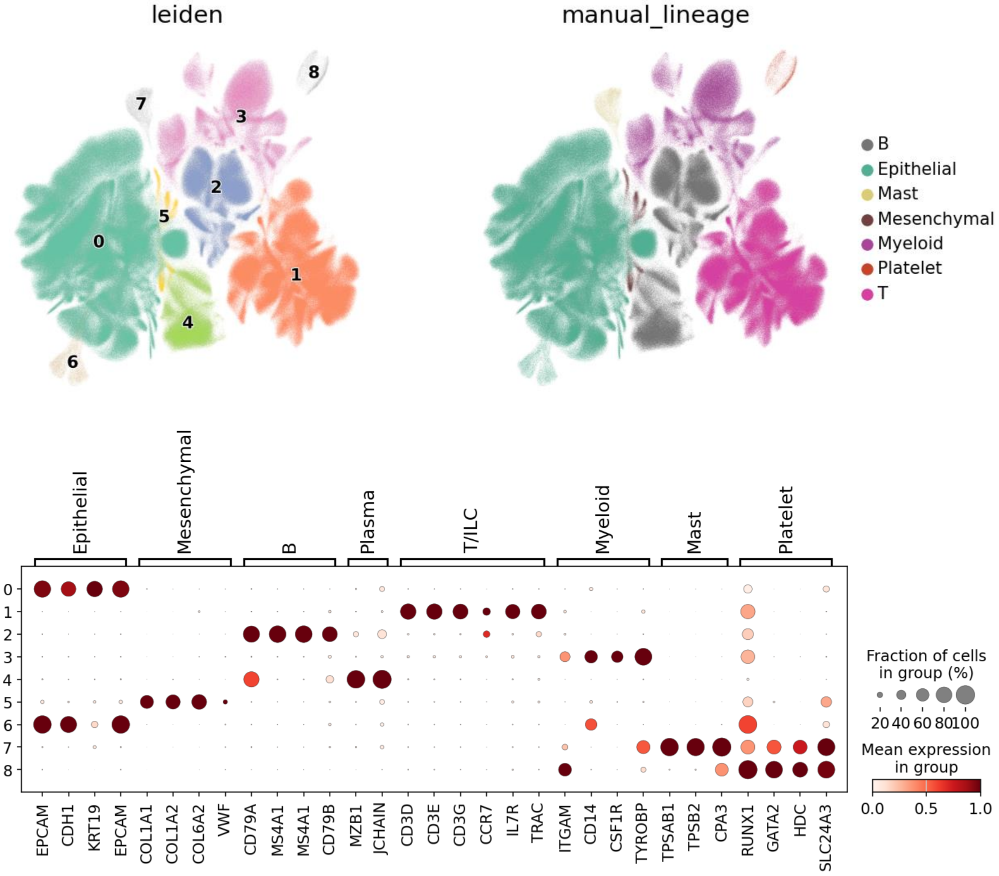

Supplementary Figure 12: **Identification of major lineages following round 1 of quality control and integration.** Leiden clusters identified by clustering at resolution of 0.03 (upper left) following the first round of quality control and integration (see Supplementary Figure S1). Expression of canonical marker genes across leiden clusters, markers derived from literature (bottom). Annotation of the major lineages after grouping (upper right).

medRxiv preprint doi: https://doi.org/10.1101/2025.06.24.25330216; this version posted June 25, 2025. The copyright holder for this preprint (which was not certified by peer review) is the author/funder, who has granted medRxiv a license to display the preprint in perpetuity.
It is made available under a CC-BY 4.0 International license .

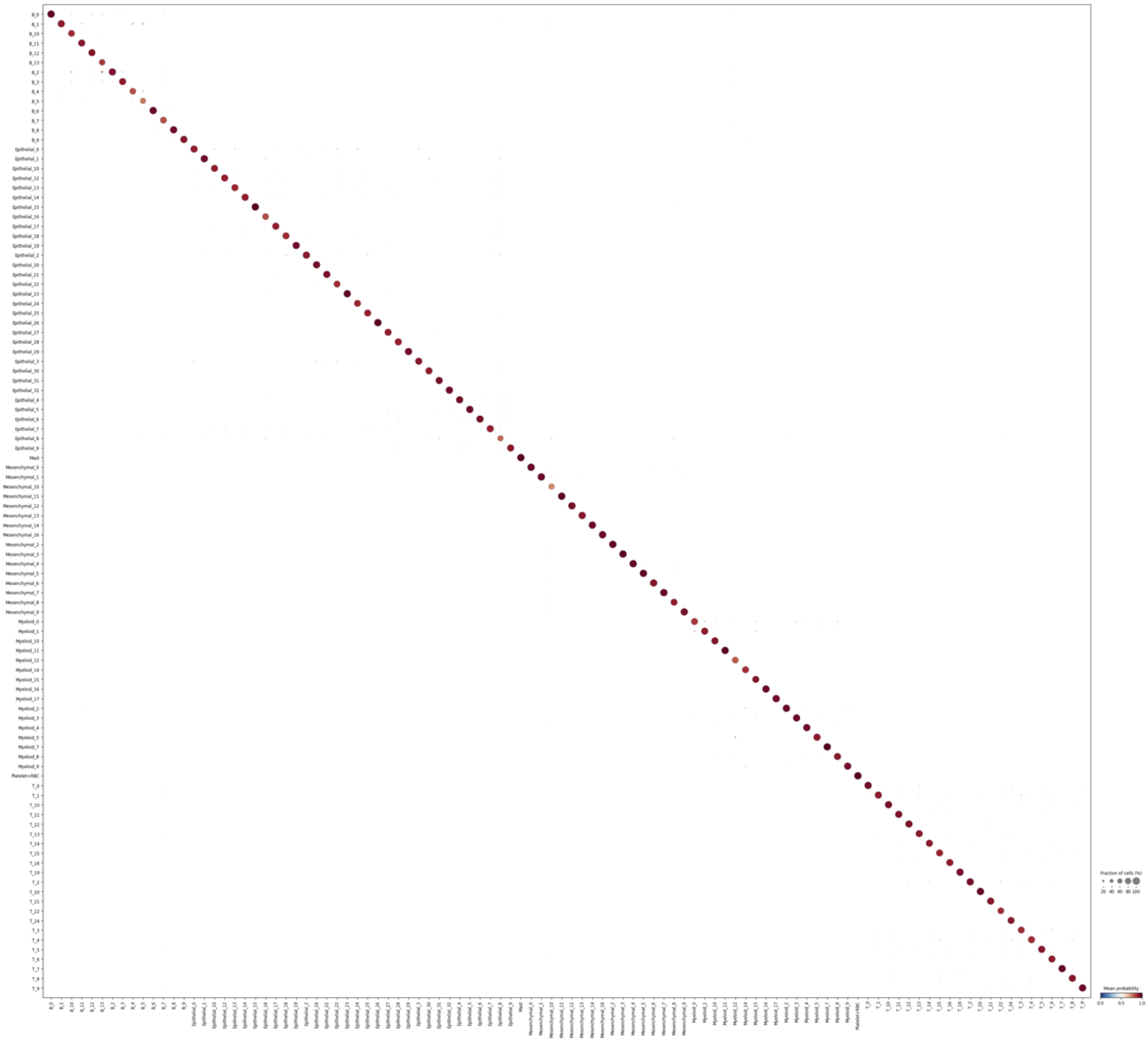

Supplementary Figure 13: **Accuracy of autoannotation model.** Comparison of the original cluster labels (x-axis) with the CellTypist model predicted labels (y-axis) for cells in the original atlassing dataset.

medRxiv preprint doi: https://doi.org/10.1101/2025.06.24.25330216; this version posted June 25, 2025. The copyright holder for this preprint (which was not certified by peer review) is the author/funder, who has granted medRxiv a license to display the preprint in perpetuity.
It is made available under a CC-BY 4.0 International license .

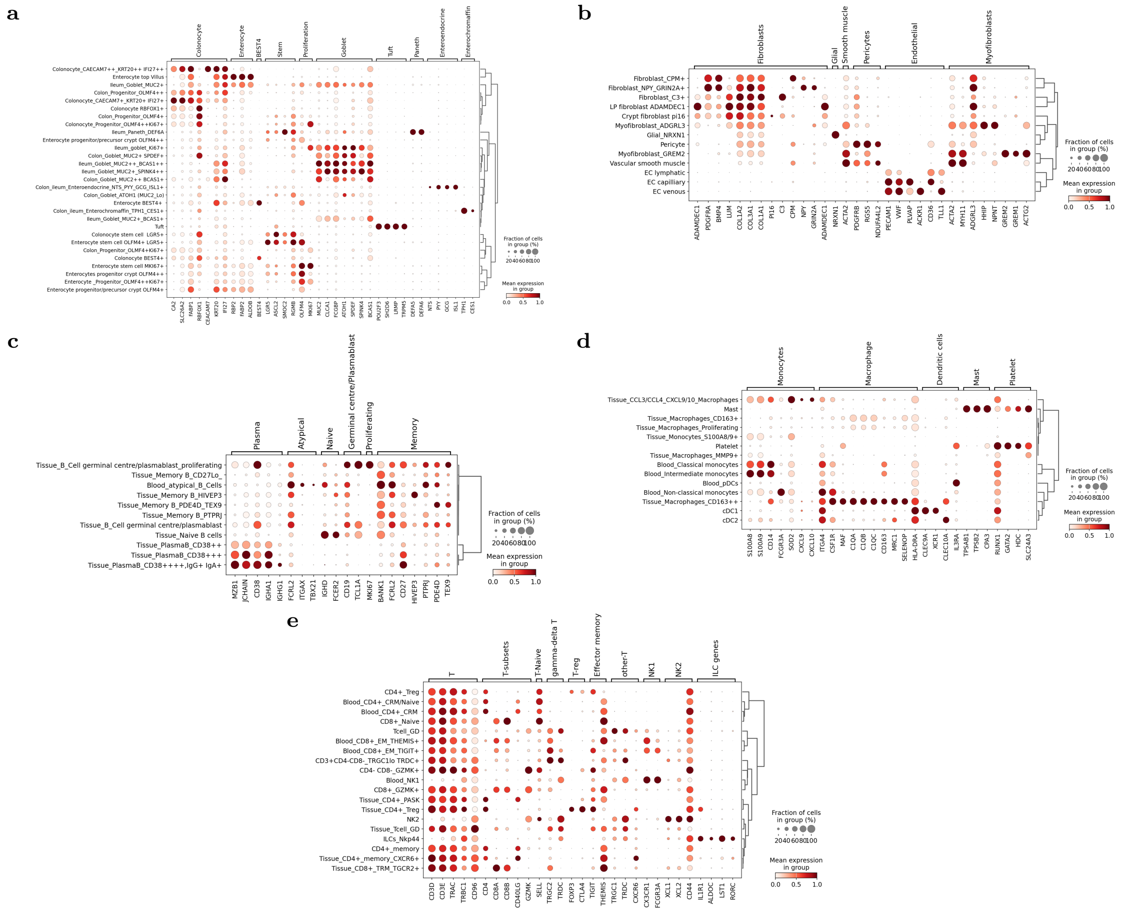

Supplementary Figure 14: **Marker gene expression for the cellular annotations.** a-e) Dotplots for the expression of demarcating and discriminatory genes used to annotate clusters from each lineage, defined as in Supplementary Figure S12.
